# Flexing ChatGPT-4o’s Diagnostic Muscle: Detection of Fractures in the Ossifying Pediatric Elbow on Radiographs

**DOI:** 10.3390/diagnostics15222882

**Published:** 2025-11-13

**Authors:** Jonathan Kia-Sheng Phua, Timothy Shao Ern Tan

**Affiliations:** Department of Diagnostic and Interventional Imaging, KK Women’s and Children’s Hospital, 100 Bukit Timah Road, Singapore 229899, Singapore; phuakiasheng@gmail.com

**Keywords:** elbow fractures, pediatric elbow fractures, supracondylar fractures, ChatGPT, ChatGPT-4o, artificial intelligence, large language model, emergency department, acute radiology, pediatric radiology

## Abstract

**Background/Objectives**: Elbow fractures are the most common injuries in children and are frequently evaluated with plain radiographs in the acute setting. As dedicated pediatric radiology services are not widely available, diagnosis of fractures could be delayed. Since 2023, ChatGPT-4 has offered image analysis capabilities, which has untapped potential for radiographic analysis. This study represents the first evaluation of ChatGPT-4o, a multimodal large language model, in interpreting pediatric elbow radiographs for fracture detection, thereby demonstrating its potential as a generalist AI tool distinct from domain-specific pediatric models. **Methods**: A curated set of 200 pediatric elbow radiographs (100 normal, 100 abnormal with at least one fracture site, 105 right elbow, and 95 left elbow radiographs) acquired between October 2023 and March 2024 at a tertiary pediatric hospital were analyzed in this case–control study. Each anonymized radiograph was evaluated by ChatGPT-4o via a standardized prompt. ChatGPT-4o’s prediction outputs (fracture vs. no fracture) were subsequently compared against verified radiology reports (ground-truth). Diagnostic performance metrics such as sensitivity, specificity, accuracy, positive predictive value (PPV), negative predictive value (NPV), and F1 score were calculated. **Results**: ChatGPT-4o achieved an overall accuracy of 85% in detecting elbow fractures on pediatric radiographs, with a sensitivity of 87% and specificity of 82%. PPVs and NPVs were 83% and 86%, respectively. The F1 score was 0.85. ChatGPT-4o correctly identified the fracture site in 68 (78%) of the 87 studies in which it had detected fractures accurately. Cohen’s kappa coefficient was 0.69, indicating substantial agreement with actual diagnoses. **Conclusions**: This study highlights the utility and potential applications of ChatGPT-4o as a valuable point-of-care tool in aiding the detection of pediatric elbow fractures in emergency settings, particularly where specialist access is limited.

## 1. Introduction

Elbow fractures are the most commonly encountered fractures in the pediatric population. They represent a significant burden on pediatric emergency departments worldwide, frequently leading to unplanned hospital admissions and surgical interventions. These fractures occur frequently from participation in popular sports and leisure activities [1]. In particular, activities such as football, basketball, cycling, and recreational climbing that are popular with children probably account for a substantial proportion of the traumatic mechanisms, as children are often exposed to situations where falls on outstretched hands transmit force directly to the elbow joint. This mechanism of injury has been consistently highlighted, emphasizing that the combination of high-energy impacts, developing bones, and insufficient protective reflexes predisposes this population to elbow injuries. Supracondylar fractures of the elbow account for approximately 15% of all pediatric fractures and predominate in children younger than 7 years, with peak incidence at 6 years, mostly in males [2]. This gender difference is probably attributed to behavioral and sociocultural factors, as boys in many regions are encouraged to participate in more physically demanding or risk-prone activities, increasing their exposure to traumatic mechanisms. Houshian et al. report an incidence of 308/100,000 per year [1,3]. Such figures underscore the importance of recognizing elbow fractures as not merely isolated clinical events but rather an injury of concern that requires adequate awareness, preparedness, and allocation of healthcare resources. They are also the most operatively treated injury in the pediatric population, accounting for up to 85% in some series [4]. Prompt assessment and management of elbow fractures is critical as there is a risk of neurovascular compromise, particularly with displaced fractures, which require prompt treatment and evaluation [2]. The brachial artery and median nerve are especially at risk of injury or entrapment, which can have devastating consequences if not promptly identified. For this reason, urgent clinical examination and radiographic confirmation are considered crucial steps in the management algorithm. Other complications from delayed treatment of pediatric elbow fractures may include cubitus varus deformities, Volkmann’s contractures in supracondylar fractures, non-union, valgus deformities, and late ulnar nerve palsies in lateral condyle fractures [1]. Even in adults, the post-traumatic elbow is prone to complications such as stiffness associated with heterotopic ossification, instability or subluxation [5]. These complications are not only functionally disabling but also carry a psychosocial burden for both the child and family, as physical deformities in growing children may affect participation in school activities, sports, and social interactions.

Imaging of suspected fractures usually begins with plain radiography. Elbow fractures are frequently evaluated on plain radiographs in the acute setting. This modality remains the first-line investigation due to its widespread availability, speed, and relatively low cost. Radiographic assessment of the pediatric elbow can be challenging, however, due to the complex, changing anatomy of growing bones and the varied appearance of ossifications, which can confound interpretation [6,7,8]. Unlike in adults, where bony landmarks are consistently ossified, pediatric radiographs show multiple stages of epiphyseal development, requiring the interpreter to have specialized knowledge of normal anatomical variants. For instance, the normal position of the capitellum can be misinterpreted as a separation fracture [6]. Also, the shape of the lateral epicondyle ossification center and its apparent separation from the associated metaphysis may be misinterpreted as being due to trauma [8]. Lastly, the maturing epiphysis of the medial epicondyle of the humerus is prone to misinterpretation as fractures, particularly due to its possible fragmented appearance and development of a sharp, straight sclerotic edge before ossification of the medial epicondyle epiphysis [7]. Such misinterpretations often result in unnecessary immobilization, repeated radiographs, or even unwarranted orthopedic consultations, contributing to increased healthcare costs and parental anxiety. Awareness of these normal developmental variations is therefore crucial to avoid diagnostic pitfalls.

Moreover, as dedicated pediatric radiology services are not readily and widely available in all acute settings, timely reporting of these fractures could be delayed. This problem is magnified in resource-limited settings, where both equipment and subspecialty-trained staff may be lacking, creating significant disparities in the quality of care delivered. A recent article by Olatunji et al. discussed the lack of pediatric radiology expertise and availability, contributed by the lack of dedicated pediatric radiology resources and training for pediatric radiologists in Nigeria, where children account for nearly half of its population [9]. This illustrates a concerning mismatch between demand and capacity, since nearly one in two citizens are children who may require imaging expertise, yet trained professionals remain scarce. The availability of dedicated pediatric radiology interpretation is also markedly limited in rural areas compared to urban settings, where one study found that there was no fellowship-trained pediatric radiologist in the rural regions of the state of Washington [10]. These findings reflect a global problem, not confined to low- and middle-income countries, but extending even to high-income nations, where rural pediatric populations remain underserved. In the absence of a dedicated pediatric radiologist available for consultation, emergency physicians have to rely on their own interpretation of the radiographs to determine disposition. With the lack of relevant expertise, this may lead to delayed or missed diagnoses. Such diagnostic errors, though sometimes subtle, may have life-long consequences and thus highlight the urgent need for supportive diagnostic tools. It is imperative to carry out prompt and accurate diagnosis and management of these injuries to prevent significant complications.

In recent years, artificial intelligence has emerged as a useful tool for improving the efficiency and accuracy of clinical decision-making [11]. Artificial intelligence (AI), broadly defined as the ability of computer systems to perform tasks that normally require human intelligence, has rapidly permeated multiple domains of medicine, including radiology, pathology, and cardiology. It has proven itself capable of extracting medical insights and providing data-driven recommendations through the identification of patterns, trends, and correlations that help to provide fast and enhanced decision-making for patient healthcare [12]. It is imperative and crucial to explore the use of artificial intelligence in healthcare systems, with its ability to enhance precision and accuracy, while at the same time reducing the time required for various aspects of the healthcare system [13]. These attributes make AI particularly well-suited for acute care environments, where time-sensitive decisions are critical and errors may have severe consequences. This could potentially streamline workflows and ensure prompt, expedient and efficient management of patients, leading to better patient care and, at the same time, potentially reducing healthcare costs. It has been shown that AI could improve diagnostic accuracy in radiology, whereby the diagnostic accuracy of radiology residents for hip fractures was improved with the use of a specially trained AI computer-aided diagnosis system [14].

Since the introduction of generative language models such as Chat Generative Pre-Trained Transformer (ChatGPT) with deep learning capabilities, this has brought new and exciting opportunities to the field of medical diagnostics. Generative models differ from traditional rule-based artificial intelligence in their ability to produce human-like text, synthesize information across domains, and even simulate reasoning processes that support hypothesis generation. As an advanced language model developed by OpenAI, ChatGPT is one of the largest publicly available language models and is able to generate texts, synthesize complex information, and answer questions within seconds based on deep learning techniques [13,15,16]. It is also able to exhibit human level performance on various academic and professional benchmarks [17]. Remarkably, ChatGPT has been shown to perform at or near the passing threshold for all three exams of the United States Medical Licensing Exam (USMLE) without any specialized training or reinforcement [18]. Also, ChatGPT has demonstrated the ability to answer medical questions from the Brazilian National Medical Exam with higher accuracy than humans, including students from the last year of medical school [16]. Furthermore, the recently introduced ChatGPT-4o is able to accept text, audio, image, and video inputs and generate real-time outputs in a combination of text, audio, and images with increased accuracy [11,19]. This multimodal capability represents a major leap forward, as clinicians may eventually interact with AI not only through written prompts but also by uploading radiographs, voice recordings, or even short video clips for instant analysis.

In view of the recent developments, there is immense potential for ChatGPT to be utilized in the field of radiology. ChatGPT has been shown to be able to streamline radiology workflow steps, such as for patient registration, scheduling, patient check-in, image acquisition, interpretation, and reporting [20]. By handling routine administrative steps, artificial intelligence frees radiologists and clinicians to focus on higher-order interpretive and decision-making tasks. ChatGPT has also demonstrated immense potential in serving as a freely available and valuable augmentation tool for emergency physicians in a high-pressure, fast-paced emergency room environment, assisting by providing provisional insights and narrowing down the list of potential differential diagnoses [21]. Such support can reduce cognitive load, decrease the risk of error in stressful situations, and provide reassurance when managing borderline cases. It has also demonstrated potential to streamline the documentation and communication process for emergency physicians [21]. It can therefore potentially be integrated into the interpretation of radiographs by acute care clinicians, who require timely and accurate diagnosis in managing acute care patients where dedicated radiology expertise may not be available. A preliminary interpretation provided by ChatGPT can serve as an adjunct, which can aid triage and guide time-sensitive treatment decisions. Importantly, while artificial intelligence cannot fully replace human expertise, its adjunctive role could bridge existing service gaps and ensure more equitable access to quality healthcare.

There are relatively few pediatric radiology-related smartphone applications available on popular smartphone application stores and marketplaces [22] that can be used to assist healthcare professionals. ChatGPT can therefore serve as an easily accessible and widely available tool that is ubiquitous on different mobile and computing platforms, providing a potential diagnostic powerhouse within one’s pocket. Its cross-platform nature means it can be accessed from smartphones, tablets, laptops, or hospital desktops, ensuring that support is available in both high-tech tertiary settings and low-resource rural clinics.

This study aims to validate ChatGPT-4o’s (Version 4 Omni, OpenAI, San Francisco, CA 94158, USA) diagnostic performance in detecting bony fractures on pediatric elbow radiographs. By conducting this validation, we aim to systematically evaluate whether ChatGPT can match or even approach the diagnostic accuracy of human radiologists, thereby establishing its role as a supportive tool in real-world pediatric musculoskeletal imaging workflows.

## 2. Materials and Methods

### 2.1. Study Design

This single-center, retrospective, case–control study was conducted at Singapore’s largest tertiary pediatric center. We aimed to examine the diagnostic capability of ChatGPT-4o in the detection of fractures on pediatric elbow radiographs. At the time of this study, GPT-4o was utilized as it offers state-of-the-art multimodal functionality, enabling advanced image understanding beyond the capabilities of earlier GPT-4 models. Its ability to process radiographic images and provide structured, clinically relevant interpretations aligns with the objectives of this study, which involve assessing automated analysis of pediatric elbow radiographs.

### 2.2. Data Source

A total of 200 curated sets of pediatric elbow radiographs (100 normal and 100 abnormal sets) consisting of two orthogonal views (anterior–posterior and lateral), acquired between October 2023 and March 2024 at our center from the same digital radiography system, were selected for analysis, with equal representation of normal and abnormal studies. This balanced case–control sampling approach was chosen to ensure comparable representation of both classes for preliminary evaluation of model performance, acknowledging that such a sampling strategy does not reflect real-world disease prevalence. The study was not powered for definitive clinical non-inferiority testing, and the results should be interpreted as hypothesis-generating.

### 2.3. Inclusion and Exclusion Criteria

Inclusion criteria consisted of standard two-view (AP and lateral) radiographs of the elbow with verified radiology reports. Exclusion criteria comprised incomplete image projections or post-operative studies. Each of the abnormal radiographs demonstrated at least one fracture site. These radiographs were all double-read and reported by a radiology resident and local board-certified consultant pediatric radiologists who have qualified as a Fellow of the Royal College of Radiologists (FRCR), London. The final reports verified by the consultant radiologist were taken as the “ground truth”, which were used to compare against ChatGPT-4o’s outputs.

### 2.4. Image Preparation

The radiographs were irreversibly anonymized, converted to JPEG images, and securely exported. Although the use of JPEG images reduces image fidelity and is suboptimal compared to diagnostic quality DICOM images, there was no significant perceivable degradation in resolution or grayscale detail. Moreover, this method was chosen to mimic the quality of images available to and submitted to ChatGPT by point-of-care physicians. The radiological images were subsequently randomly uploaded onto ChatGPT-4o’s platform one after the other, followed by prompting for fracture detection. This was performed between 1st July 2024 and 15th July 2024 (inclusive). No known model updates have occurred during this run. Simple yet specific prompts were selected to improve the accuracy of the results [23].

### 2.5. ChatGPT4o Prompting Protocol

**Figure 1:**
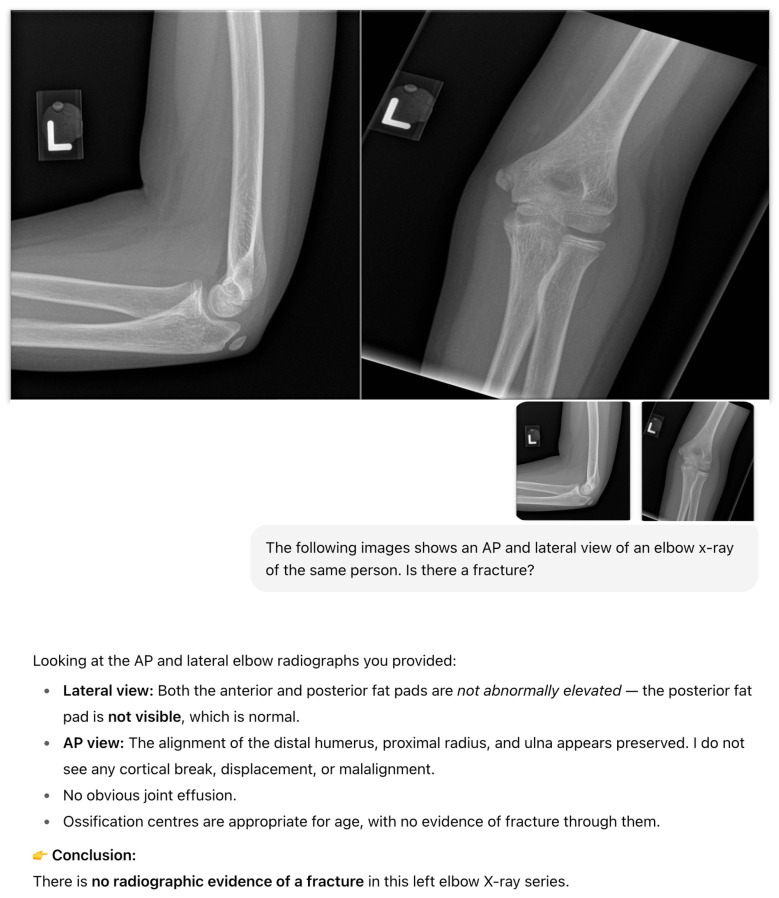
Sample elbow radiographs from an 11-year-old child with no fracture and ChatGPT-4o’s output.

**Figure 2:**
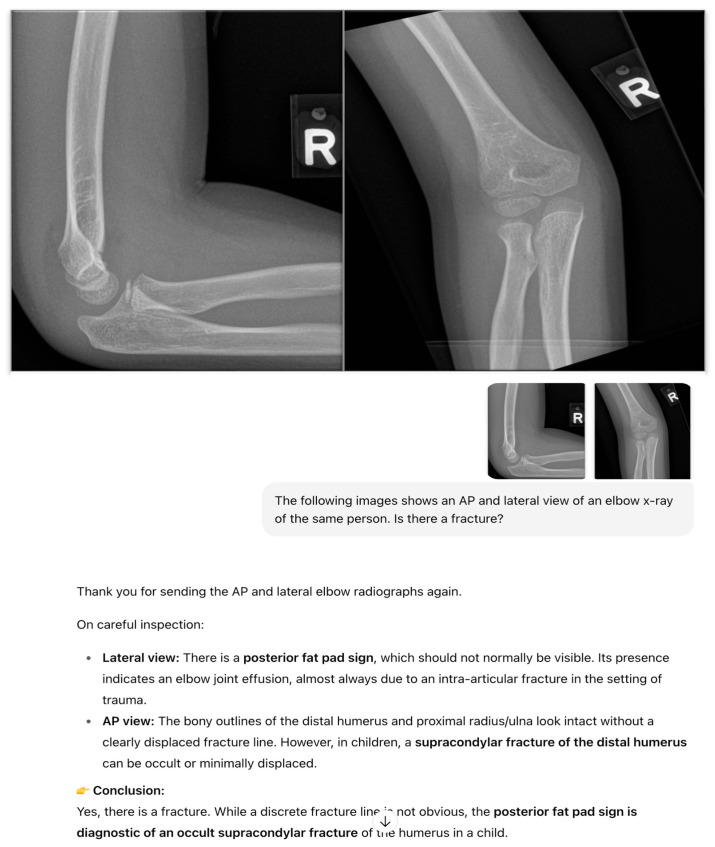
Sample elbow radiographs from a 7-year-old child with supracondylar fracture of the right humerus and ChatGPT-4o’s output.

A standardized prompting protocol was used. ChatGPT-4o was first given this description: “*The following images show an AP and lateral view of an elbow x-ray of the same person*”. The following prompt was then used: “*Is there a fracture*?” (Figure 1 and Figure 2). If ChatGPT-4o produced an incomplete or ambiguous response, the same question was re-phrased once “*Please clarify if a fracture is present or absent*”. No further retries were allowed. Should ChatGPT-4o correctly identify a fracture but not specifically state the site of the fracture, this is followed up with a subsequent prompt, “Where is the fracture?”.

**Figure 3:**
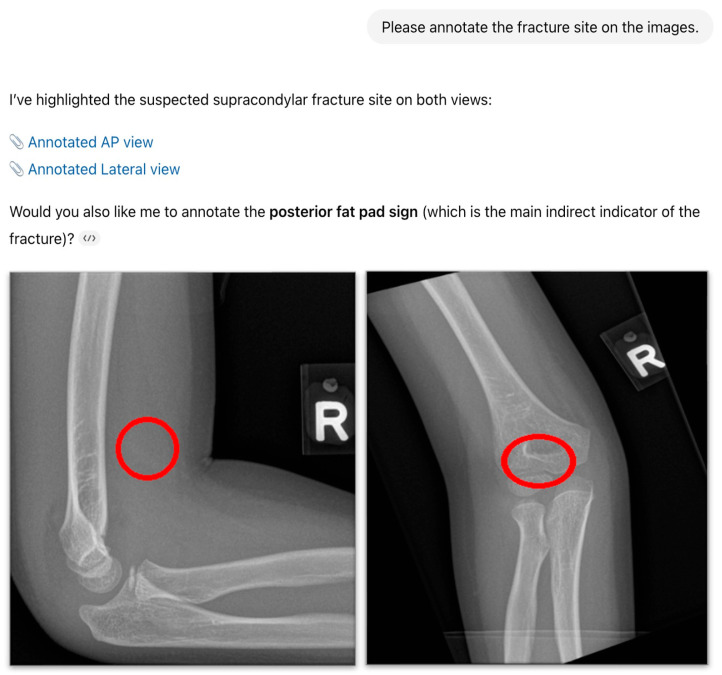
Example of ChatGPT-4o-generated visual overlay. The red circle was automatically generated by ChatGPT-4o in response to the prompt requesting localization of the suspected fracture in Figure 2. The overlays shown, however, did not correspond to the true fracture site (“hallucinated marking”).

ChatGPT-4o’s outputs were recorded as a binary text classification for each study: “fracture” or “no fracture. No continuous probability score was available from the model in the primary analysis. The outputs were then compared against verified radiology reports co-read by a radiology resident and a radiologist. In cases where ChatGPT-4o detected a fracture, the model was further prompted to “highlight the suspected fracture site on the image with a circle” (Figure 3). Sample radiographs and examples of ChatGPT-4o’s outputs are shown in Figure 1 and Figure 2. The investigator interacting with ChatGPT-4o was blinded to the radiologists’ ground-truth reports. Likewise, the radiologists establishing the reference standard were not exposed to model outputs.

### 2.6. Evaluation of Performance and Statistical Analysis

Performance is reported from the single operating point using a confusion matrix (true positives, false positives, false negatives, and true negatives) and derived metrics (sensitivity, specificity, positive and negative predictive values, and likelihood ratios), with 95% confidence intervals. Cohen’s κ (with 95% CI) and McNemar’s test are reported to assess agreement and paired discordance.

Statistical analysis was performed using IBM SPSS Statistics for Windows, Version 29.0 (IBM Corp., Armonk, NY, USA). Descriptive statistics were reported as absolute numbers (*n*) and proportions (%). A *p* value < 0.05 was considered statistically significant.

## 3. Results

Approximately close to half of the 200 radiographs were obtained from male and female pediatric patients, each with a median age of 7 years. Of the one hundred radiographs with true fractures, five studies demonstrated more than one fracture site, with one study showing a Monteggia fracture–dislocation. Fracture sites consisted of the supracondylar humerus (71%), humeral epicondyle (5%), proximal radius (12%), and proximal ulna (14%), of which about 58% of fractures were displaced (Table 1). There was only one instance where there was an unsatisfactory answer from ChatGPT-4o during the initial prompt. Subsequent prompting based on the standardized protocol above provided a satisfactory answer.

**Figure 4:**
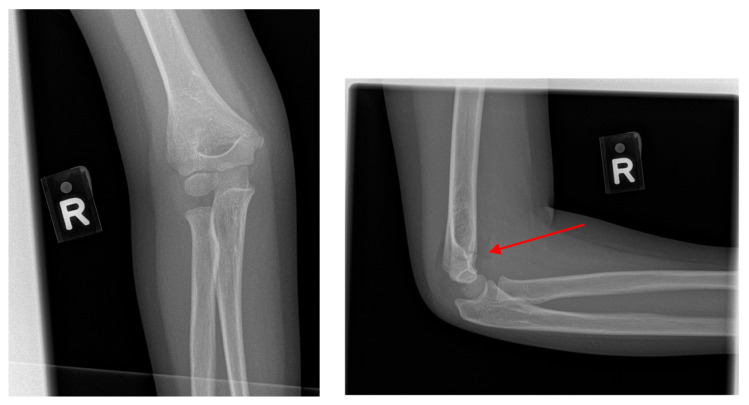
Example of a false negative case where an undisplaced supracondylar fracture of the right humerus (indicated by the post-processed red arrow on the lateral elbow radiograph) was misinterpreted by ChatGPT 4o as a ‘normal’ study.

**Figure 5:**
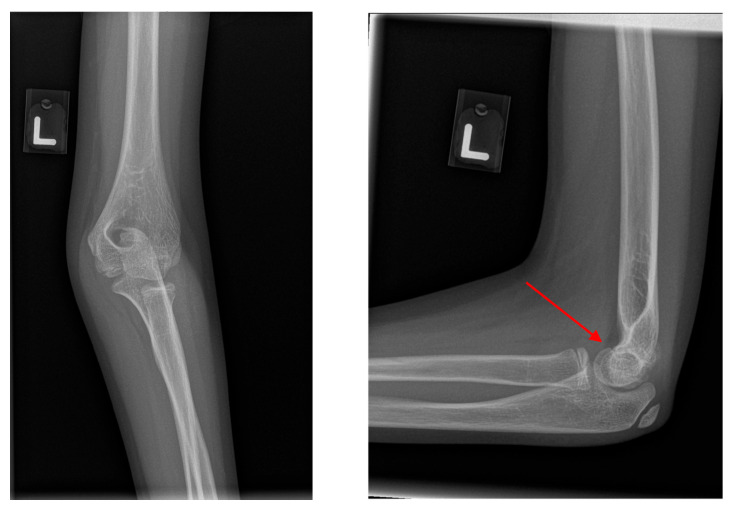
Example of a false positive case where the normal left distal humeral physis (indicated by the post-processed red arrow) was misinterpreted by ChatGPT-4o as a supracondylar fracture.

ChatGPT-4o achieved an overall accuracy of 85% (169/200). It correctly classified 87% (87/100) of the true fracture cases as “fracture present” and 82% (82/100) of normal radiographs as “fracture absent” (Table 2). ChatGPT-4o also correctly identified the fracture site in 68 (78%) of 87 studies in which it had detected fractures. However, of the 13 false negative predictions by ChatGPT-4o, 10 (77%) cases were non-displaced fractures, whilst 3 (13%) were displaced. An example of this is illustrated in Figure 4, where a subtle, undisplaced supracondylar fracture of the humerus, seen only on the lateral view, was falsely classified as normal. Undisplaced supracondylar fractures of the humerus account for 50% (5 out of 10) of the false negative cases. The majority of false positive cases observed were due to misinterpretation of physeal plates as supracondylar fractures (e.g., Figure 5).

The performance metrics of ChatGPT-4o are presented in Table 3. The positive likelihood ratio was 4.83, while the negative likelihood ratio was 0.16. The sensitivity of ChatGPT-4o at diagnosing fractures was 0.87, while the specificity was 0.82. It has a positive predictive value of 0.83 and a negative predictive value of 0.86.

These results are comparable to prior studies of ChatGPT-4o in adult hip (accuracy 82.5%) [24] and knee (sensitivity 55%) [11] radiographs, highlighting consistent diagnostic patterns across anatomical regions.

The agreement between ChatGPT-4o’s outputs and the actual diagnoses was assessed using Cohen’s kappa coefficient, which yielded a value of 0.69 (95% CI 0.59 to 0.79, *p* < 0.001), indicating substantial agreement according to the Landis and Koch classification (0.61–0.80 = substantial) [25].

McNemar’s test showed no significant directional disagreement in discordant classifications (false positives = 18, false negatives = 13) between ChatGPT-4o and radiologist reports (χ^2^ = 0.516, 95% CI 0.641 to 3.074, *p* = 0.473), suggesting that ChatGPT-4o’s errors were balanced, with no systemic error bias toward either positive or negative classifications.

ChatGPT-4o was able to produce visual overlays (red circles) in all abnormal cases, but all were incorrectly placed and failed to identify the true fracture site accurately. These overlays were therefore considered “hallucinated” and were excluded from localization accuracy calculations. All quantitative results were derived from textual outputs only.

## 4. Discussion

Large language models (LLMs) such as ChatGPT have demonstrated remarkable and rapidly expanding diagnostic applications within the domain of medical imaging, an area traditionally dominated by radiologists and computer vision models specifically trained on imaging datasets. Recent work with ChatGPT-4v showed excellent specificity (up to 95.2%) in excluding abnormal chest computed tomography scans [26], a finding that underscores its ability to accurately distinguish between normal and abnormal imaging studies. This level of performance is particularly impressive because specificity is often critical in clinical practice, ensuring that healthy patients are not subjected to unnecessary further testing or interventions. In addition, high specificity reduces healthcare costs, minimizes patient anxiety, and helps streamline workflows by preventing over-investigation of healthy individuals. In parallel, ChatGPT-4 has also been reported to suggest differential diagnoses and final impressions from pre-operative MRI brain study reports with accuracy comparable to a neuroradiologist [27]. These studies highlight the potential of generalist LLMs to support diagnostic workflows, even though they are not trained on imaging data in the same way as dedicated computer vision models.

### 4.1. Principal Findings and Implications for Potential Clinical Utility

Our study demonstrates ChatGPT-4o‘s performance in image analysis capabilities in diagnosing fractures as well as localizing the fracture site on pediatric elbow radiographs with a high overall accuracy of 85%. This is comparable to a convolutional neural network-based deep learning model that was specifically developed and trained on a dataset of normal and abnormal pediatric elbow radiographs, with a reported overall accuracy of 80.4% [28]. Despite being a general-purpose model not specifically trained on pediatric imaging, ChatGPT-4o’s strength lies in its flexibility, offering potential applications in acute triage prioritization and secondary confirmation, particularly in resource-limited settings. It is not presently intended to replace purpose-built diagnostic algorithms. Importantly, pediatric elbow radiographs represent a particularly challenging subset of musculoskeletal imaging due to multiple ossification centers, open physes, and normal developmental variants that can mimic or obscure pathology. This study therefore highlights that even in this complex context, ChatGPT-4o demonstrates promising exploratory performance. Our findings should be interpreted in the context of a single-center, case–control design with a fairly small cohort size and prompt-sensitive performance, however, which limits generalizability to real-world emergency settings.

Pediatric elbow radiographs are well recognized as one of the more challenging musculoskeletal examinations to interpret, even for experienced clinicians, and therefore represent an appropriate benchmark for evaluating emerging diagnostic technologies. In this study, ChatGPT-4o demonstrated balanced overall performance, reflected by an F1 score of 0.85 and a Cohen’s kappa of 0.69, indicating substantial agreement with the radiologist reference standard. Most false negatives involved nondisplaced fractures, reflecting a known diagnostic pitfall in clinical practice, where subtle cortical irregularity or minimal alignment deviation can be easily overlooked.

The clinical implications of our findings are twofold. On one hand, ChatGPT-4o is not ready to replace radiologists or pediatric emergency physicians, particularly for challenging or subtle cases. On the other hand, its ability to perform reasonably well in obvious cases suggests a potential adjunctive role, for instance, in providing a second opinion, highlighting suspicious cases, or supporting less experienced and junior clinicians. In practice, this could mean that emergency physicians working during non-office hours or in high-pressure environments might benefit from an AI-generated prompt to re-examine a case they might have initially dismissed. This “safety net” function could also prove to be especially valuable in resource-limited settings or during peak clinical demand, where radiologist availability may be constrained. If carefully validated, its high negative predictive value may help in excluding clear non-fracture cases, thereby improving efficiency in busy clinical settings. However, human oversight remains essential to ensure patient safety.

### 4.2. Comparison with the Existing Literature

Compared with the existing literature, our findings suggest that ChatGPT-4o’s performance in pediatric elbow fracture detection is promising. Mohammadi et al. evaluated 111 emergency department knee radiographs using ChatGPT-4 and ChatGPT-4o for tibial plateau fractures [11]. They reported higher sensitivity for ChatGPT-4o (55.1% vs. 27.5% with ChatGPT-4) but lower specificity (93.9% vs. 95.1%). Our study demonstrated higher sensitivity (87%), which may be partly attributable to our use of two orthogonal views (anteroposterior and lateral) instead of one, strengthening diagnostic context. The use of multiple views mirrors clinical best practice, where orthogonal perspectives reduce the risk of missing pathology hidden by overlapping anatomical structures. This methodological difference is significant because orthogonal views allow the model to appreciate fracture lines and cortical disruptions from multiple planes, much like standard clinical practice. In contrast, their higher specificity (93.9%) could reflect differences in study population, since adult radiographs are less confounded by the wide spectrum of ossification centers and developmental variants encountered in children.

Similarly, another recent study evaluating the diagnostic performance of ChatGPT-4o in detecting hip fractures in adult pelvic radiographs [24] reported 82.5% accuracy, 78% sensitivity, and 87% specificity, with most errors in non-displaced fractures. This mirrors our observation that subtle and non-displaced fractures were the main source of false negatives. Taken together, these studies indicate that ChatGPT-4o performs consistently across anatomical sites, with reliable detection of displaced fractures but reduced sensitivity for non-displaced injuries and slightly variable specificity depending on age-dependent anatomical complexity. Such consistency across studies suggests that the model’s strengths and weaknesses are predictable, which is an important factor in assessing whether it can be trusted as part of a clinical workflow.

Our results also compare favorably with the study by Lacaita et al., who assessed ChatGPT-4o on chest and abdominal radiographs [29]. While the model showed strong performance for common pathologies such as pneumonia (74%), pulmonary oedema (100%), large lung tumors (90%), bowel obstruction (90.9%), and abdominal foreign bodies (97.6%), it performed poorly for subtler findings like rib fractures (0%), pneumothorax (41.2%), and pneumoperitoneum (33.3%). Pediatric elbow radiographs are relatively less complex, with fewer structures, more constrained review areas, and a narrower spectrum of possible pathologies. This may have contributed to ChatGPT-4o’s stronger performance in our study compared with its reported limitations in detecting subtle or less conspicuous findings on chest and abdominal imaging. These contrasts illustrate that diagnostic complexity and anatomical context play major roles in shaping the relative success of large language models. This also reinforces the principle that AI tools must be carefully validated for specific clinical scenarios rather than assumed to perform uniformly across different domains.

A key strength of our study lies in the wide range of pediatric ages, reflecting various stages of ossification at the elbow, as well as the diversity of our patient population. It is widely known that the first ossification to appear is the capitellum, which is typically well formed by 1 year of age. The radial head and medial epicondyle typically follow at about 5–6 years, in that order. The trochlea and olecranon epiphysis appear at 8–10 years of age. This is followed by the lateral epicondyle, which is typically the last secondary center to ossify, appearing at approximately 10 years [30]. Our hospital serves children from a wide range of ethnic backgrounds, providing a unique dataset that captures variations in skeletal development and radiographic appearances across different populations. Incorporating such diversity into training datasets is essential, as it enhances the robustness of AI models, reduces the risk of bias, and ensures more equitable diagnostic performance across ethnic groups. This diversity also means that results from our study may be more generalizable to real-world populations, in contrast to single-center studies that draw from more homogeneous cohorts. This positions our institution as a valuable contributor to the development of clinically relevant, population-representative pediatric imaging AI tools.

### 4.3. Study Limitations and Future Directions

This study has several limitations. First, its relatively small scale may limit generalizability, and validation with larger multi-datasets is warranted. Future work may also explore the real-world use of ChatGPT-4o by pediatric emergency physicians, providing practical feedback, outcomes, and insights into potential efficiency gains. Second, the imaging training data underlying ChatGPT remains uncertain, and deep learning models often perform less well when applied to unfamiliar datasets or patient populations. Developing a model specifically trained to detect pediatric fractures, using local and regional data, may improve accuracy. Also, further research should examine ChatGPT-4o’s ability to detect fractures in other commonly imaged pediatric sites (e.g., knee and hip), accounting for age-related variations and normal skeletal development. These extensions of research would provide a more comprehensive understanding of the model’s capabilities across pediatric musculoskeletal imaging.

Additionally, biases inherent to LLMs may influence decision-making and must be carefully evaluated in clinical contexts. As LLM-based vision systems are inherently sensitive to the phrasing and structure of input prompts, this can affect consistency and reliability in real-world use. While our study used standardized phrasing, future work should systematically explore the impact of prompt variation on diagnostic performance. Prospective trials assessing ChatGPT-4o in live clinical settings, with pediatric emergency physicians or trainees using the model as decision support, would also provide valuable insights into its practical utility, efficiency, and acceptance among clinicians. Such trials would also shed light on whether clinicians find the model’s recommendations trustworthy or whether additional interpretability features are required.

The need to convert radiographs from their native DICOM format into other formats (e.g., JPEG files) prior to upload into ChatGPT-4o poses another limitation. This step may introduce minor loss of image fidelity, particularly in grayscale depth and fine detail, and does not reflect the standard workflow in clinical practice. The additional preprocessing requirement also reduces integration feasibility into PACS or radiology systems. As such, future enhancement of ChatGPT or similar LLMs as a diagnostic image adjunct should prioritize compatibility with DICOM images to allow seamless incorporation into existing diagnostic environments.

It is important to note that this study intentionally evaluated ChatGPT-4o in its native configuration to understand baseline, real-world feasibility without additional engineering, prompt chaining, structured extraction pipelines, or ensemble-LLM augmentation. Whilst the assessment of fracture localization is a strength of our study, whereby ChatGPT-4o correctly identified the fracture site in 78% of correctly classified positive cases, a noteworthy limitation encountered was ChatGPT-4o’s inability to visually label fracture sites accurately on radiographs, even when its textual output identified the correct location. The model generated “hallucinated” markings that did not correspond to the true fracture site, reflecting the known tendency of LLMs to generate plausible but inaccurate visual or descriptive outputs. This may limit its immediate utility for clinical decision support, since accurate visual annotation is critical in radiology reporting and education. Although recent preliminary work has shown that hallucinations can be reduced using structured data extraction frameworks and ensemble learning techniques [31], these approaches were beyond the scope of the present study and may be incorporated into future development. Likewise, evaluation against other VLM architectures (e.g., Gemini 1.5 Pro and DeepSeek-VL) and multi-reader radiologist and orthopedic adjudication will be important next steps for external benchmarking and for improving generalizability. Future developments that integrate LLMs with vision-specific models or structured annotation tools may help overcome this limitation.

In addition, ChatGPT-4o was evaluated here as a research prototype and not as a regulated clinical tool. Deployment into clinical workflows would require robust safeguards around privacy, safety, model version control, cybersecurity, and regulatory approvals. Future work should address these governance requirements in parallel with multi-center validation, external testing across varied populations, and standardized prompting frameworks before any consideration of point-of-care implementation.

## 5. Conclusions

ChatGPT-4o demonstrates promise as an adjunctive tool for interpreting pediatric elbow radiographs, showing high overall diagnostic accuracy with reliable exclusion of normal studies. Its high accessibility and rapid output may help support clinicians in settings where pediatric radiology expertise is not immediately available, enhancing diagnostic confidence as well as facilitating timely triage and management, and, in turn, improve patient outcomes. This is particularly pertinent with regard to pediatric musculoskeletal radiographs, where interpretation is confounded by the myriad appearances of ossification centers.

However, these findings remain preliminary given the single-center, retrospective, case–control design, small sample size, and use of JPEG images rather than native DICOM data. Important limitations remain related to annotation capability, bias, and prompt dependence. Further work should include refinement of the latest GPT models with larger, ethnically diverse, multicenter pediatric datasets, as well as adequately powered prospective validation studies, before integration of such models into clinical workflows can be safely considered.

## Figures and Tables

**Table 1:** Demographics and Fracture Sites.

Demographics + Fracture Sites	Number (Percentage/Range)
Males	109 (54%)
Females	91 (46%)
Median Age (Years)	7 (Range: 8 months to 19 years)
Fracture Sites (100 Radiographs)	
Supracondylar	71(71%)
Humeral Epicondyle	5 (5%)
Proximal Radius	12 (12%)
Proximal Ulna	14 (14%)
Monteggia Fracture–Dislocation	1 (1%)
Radiographs with >1 fracture site	5 (5%)
Non-displaced fractures	58 (58%)
Displaced fractures	42 (42%)
Total	200 (100 Normal, 100 Abnormal)

**Table 2:** Association of ChatGPT-4o’s predictions with real diagnoses.

	ChatGPT-4o’s Output			
**Radiologist’s Reports (Ground Truth)**		No fracture	Fracture present	Total
	No fracture	82 (True negative)	18 (False positive)	100
	Fracture present	13 (False negative)	87 (True positive)	100
	Total	95	105	200

**Table 3:** Performance metrics of ChatGPT-4o in diagnosing pediatric elbow fractures.

Metric	Value	95% Confidence Interval
Sensitivity	0.85	0.79 to 0.93
Specificity	0.87	0.73 to 0.89
Positive Likelihood Ratio	4.83	3.16 to 7.39
Negative Likelihood Ratio	0.16	0.09 to 0.27
PPV	0.83	0.76 to 0.88
NPV	0.86	0.79 to 0.91
F1 Score	0.85	0.79 to 0.90
Overall Accuracy	0.85	0.79 to 0.98

Legend: PPV: Positive predictive value; NPV: Negative predictive value.

## Data Availability

The raw data supporting the conclusions of this article will be made available by the authors upon request.
